# Association between body mass index and risk of cardiovascular disease-specific mortality among adults with hypertension in Shanghai, China

**DOI:** 10.18632/aging.202543

**Published:** 2021-02-17

**Authors:** Jing Hu, Huilin Xu, Jingjing Zhu, Jinling Zhang, Jun Li, Linli Chen, Xiaohua Liu, Guoyou Qin

**Affiliations:** 1 Department of Biostatistics, School of Public Health, Fudan University, Shanghai, People’s Republic of China; 2 Shanghai Minhang Center for Disease Control and Prevention, Shanghai, People’s Republic of China; 3 Minhang District Branch of School of Public Health, Fudan University, Shanghai, People’s Republic of China; 4 Key Lab of Health Technology Assessment, National Health Commission of the People’s Republic of China, Fudan University, Shanghai, People’s Republic of China

**Keywords:** adults, body mass index, cardiovascular disease, hypertension, mortality

## Abstract

The aim of our study was to examine the association between body mass index (BMI) and the risk of cardiovascular disease (CVD)-specific mortality among Chinese adults with hypertension by sex. This study included 212,394 adult hypertensive patients aged 20–85 years registered in the records of Minhang District during 2007–2018. Cox proportional hazards regression was performed to evaluate the association between BMI and CVD-specific mortality among Chinese adults with hypertension. There were 14,029 deaths over an average of 8.24 years (range, 0.19–11.96 years). The multivariable-adjusted hazard ratios (HRs) and 95% confidence intervals (CIs) across BMI categories (< 18.5 kg/m^2^, 18.5–24.9 kg/m^2^ [reference group], 25.0–29.9 kg/m^2^, and ≥ 30 kg/m^2^) for CVD-specific mortality were 1.37 (1.22–1.53), 1.00 (reference), 0.95 (0.90–1.01), and 1.21 (1.04–1.40) in males, and 1.44 (1.31–1.59), 1.00 (reference), 0.96 (0.91–1.01), and 1.04 (0.92–1.17) in females. A U-shaped relationship was observed between BMI and CVD-specific mortality (overall association *P*< 0.001; non-linearity *P*< 0.001). This association was attenuated in old age. This study revealed a U-shaped relationship between BMI and CVD-specific mortality among hypertensive men and women. In older people, overweight and obesity are potential factors that reduce the risk of CVD death.

## INTRODUCTION

Hypertension, also known as high blood pressure, is one of the critical public health problem in the world [1, 2]. In 2014, approximately one billion adults had hypertension [3]. The prevalence of hypertension in China continues to rise, with more than 200 million people in 2015 [4]. Indeed, hypertension is a major cause of premature deaths in China [4–6]. Several cohort studies have found a U-shaped relationship between BMI and all-cause mortality in hypertensive patients, which means that both relatively low and high BMI will increase mortality [7, 8]. Overweight and obesity are associated with various cardiovascular disease risk factors and increased cardiovascular events [9, 10]. Cardiovascular death is a leading cause of death among hypertensive patients [11–13]. However, the research on the relationship between BMI and CVD-specific mortality in a hypertensive population is relatively limited, and the conclusions from these studies are inconsistent [14]. In people with hypertension and left ventricular hypertrophy, cardiovascular death was more likely in the thin and obese groups than in the normal body weight groups [15]. However, a prospective cohort study on men with hypertension in Texas demonstrated that men with high cardiorespiratory fitness level and obese men have no greater risk of CVD-specific-mortality compared with normal body weight counterparts [16]. Among patients with the risk of cardiac failure, those with a BMI in the range of 30.0–34.9 had lower mortality than those considered to have an ideal weight. The term “obesity paradox” refers to the above observations and the increased survival among obese patients. Due to the high prevalence of hypertension and the great number of hypertensive patients in China, it is imperative to advance understanding about the association between BMI and CVD-specific mortality in this population. This will allow for appropriate weight management advice for hypertensive patients from the perspective of reducing CVD-specific mortality.

Given the limited exploration of this issue in the Chinese population, this study aimed to explore the relationship between baseline BMI and cardiovascular mortality risk in a hypertensive populations using a retrospective cohort in Shanghai, China.

## RESULTS

This study included 212,394 patients with hypertension, with an average age of 63.3 years, ranging from 20 to 85 years. A total of 14,029 hypertensive individuals, including 6,943 men and 7,086 women, died of CVD during an average follow-up period of 8.24 years or 1,643,007 person-years. Comparing between different BMI categories reveal significant differences in demographic characteristics, lifestyle, clinical characteristics, and family history of chronic disease. Compared with other BMI categories, patients classified as underweight have lower rates of concomitant diabetes, family history of chronic diseases, smoking and drinking (Table 1).

**Table 1:** Baseline characteristics according to BMI categories in people with hypertension.

**Characteristic**	**BMI (WHO category, kg/m^2^)**				**P-value**
	**<18.5** **(N=5120)**	**18.5-24.9** **(N=130530)**	**25.0-29.9** **(N=68583)**	**≥30.0** **(N=8161)**	
Age (x̄±SD, year)	69.9±10.8	64.0±10.8	61.6±10.6	60.1±11.5	<0.001
< 60 years	1022(19.9)	49739(38.1)	31808(46.4)	3838(47.0)	<0.001
≥ 60 years	4098(80.1)	80791(61.9)	36775(53.6)	4323(53.0)	
Follow-up time (years)	7.5±3.2	8.2±2.8	8.4±2.7	8.2±2.7	<0.001
Sex (%)					
Male	2096(40.9)	60655(46.5)	33123(48.3)	3164(38.8)	<0.001
Female	3024(59.1)	69875(53.5)	35460(51.7)	4997(61.2)	
SBP (x̄±SD)	155.3±15.5	154.2±14.8	155.3±15.6	157.1±16.5	<0.001
DBP(x̄±SD)	92.1±9.3	93.2±8.9	94.8±9.5	95.6±10.1	<0.001
Concomitant diabetes					
Yes	697(13.6)	21688(16.6)	13188(19.2)	1854(22.7)	<0.001
No	4423(86.4)	108842(83.4)	55395(80.8)	6307(77.3)	
CVD death					
Yes	796(15.6)	9143(7.0)	3625(5.3)	465(5.7)	<0.001
No	4324(84.4)	121387(93.0)	64958(94.7)	7696(94.3)	
Family history of hypertension (%)					
Yes	1654(32.3)	53654(41.1)	32659(47.6)	4054(49.7)	<0.001
No	3466(67.7)	76876(58.9)	35924(52.4)	4107(50.3)	
Family history of CVD (%)					
Yes	110(2.2)	3219(2.5)	2179(3.2)	330(4.0)	<0.001
No	5010(97.8)	127311(97.5)	66404(96.8)	7831(96.0)	
Family history of stroke (%)					
Yes	110(2.2)	3385(2.6)	2217(3.2)	280(3.4)	<0.001
No	5010(97.8)	127145(97.4)	66366(96.8)	7881(96.6)	
Family history of diabetes (%)					
Yes	177(3.5)	6422(4.9)	4607(6.7)	615(7.5)	<0.001
No	4943(96.5)	124108(95.1)	63976(93.3)	7546(92.5)	
Exercise (%)					
Never	2043(39.9)	41336(31.7)	22079(32.2)	2926(35.9)	<0.001
Sometimes	2054(40.1)	57742(44.2)	30191(44.0)	3487(42.7)	
Everyday	1023(20.0)	31452(24.1)	16313(23.79)	1748(21.4)	
Smoke (%)					
Yes	867(16.9)	26264(20.1)	16108(23.5)	1624(19.9)	<0.001
Never	4253(83.1)	104266(79.9)	52475(76.5)	6537(80.1)	
Drink (%)					
Never	4424(86.4)	104320(79.9)	52218(76.1)	6546(80.2)	<0.001
Sometimes	430(8.4)	17304(13.3)	10332(15.1)	978(12.0)	
Always	266(5.2)	8906(6.8)	6033(8.8)	637(7.8)	

Abbreviations: BMI: body mass index; SD: standard deviation; SBP: systolic blood pressure; DBP: diastolic blood pressure; CVD: cardiovascular disease.

The HRs and 95% CIs across BMI categories for CVD-specific mortality were 1.37 (1.22–1.53), 1.00 (reference), 0.95 (0.90–1.01), and 1.21 (1.04–1.40) in men with a BMI of < 18.5, 18.5–24.9, 25–29.9, or ≥ 30 kg/m^2^, respectively. The corresponding HRs for women were 1.44 (1.31–1.59), 1.00 (reference), 0.96 (0.91–1.01), and 1.04 (0.92–1.17). Compared with individuals with a baseline BMI of 18.5 to 24.9 kg/m^2^, women that were under-weight and men that were under-weight or obese had increased CVD-specific mortality (Table 2).

**Table 2:** Hazard ratio and 95% confidence intervals for CVD-specific mortality of different sex in people with hypertension according to BMI category.

	**BMI(kg/m^2^)**				**Overall**	**P-value**
	**<18.5** **(N=5120)**	**18.5-24.9** **(N=130530)**	**25.0-29.9** **(N=68583)**	**≥30.0** **(N=8161)**		
Male	2096	60655	33123	3164	99038	
Deaths, n	332	4645	1784	182	6943	
Person-y	15065	485143	271914	25151	797272	
Mortality/100,000 person-y	2204	957	656	724	871	< 0.01
HR(95% CI)*	1.37(1.22-1.53)	1.00	0.95(0.90-1.01)	1.21(1.04-1.40)		
Female	3024	69875	35460	4997	113356	
Deaths, n	464	4498	1841	283	7086	
Person-y	23377	582636	304098	42155	952266	
Mortality/100,000 person-y	1985	772	605	671	744	< 0.01
HR(95% CI)*	1.44(1.31-1.59)	1	0.96(0.91-1.01)	1.04(0.92-1.17)		

Abbreviations: CVD: cardiovascular disease; BMI: body mass index; CI: confidence interval; HR: hazard ratio.

*Adjusted for sex, age at registry, systolic blood pressure, diastolic blood pressure, concomitant diabetes, family history of diabetes, family history of hypertension, family history of stroke, family history of CVD, smoking, drinking and physical activity.

**Figure 1:**
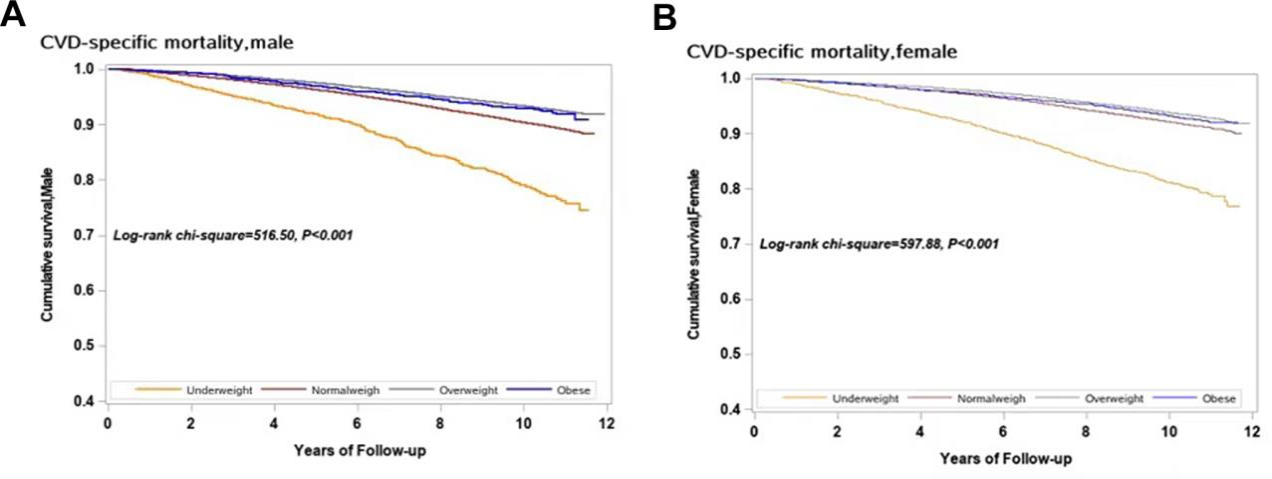
**Survival curves with regard to different categories of BMI in both sexes for CVD specific mortality.** (**A**) Male; (**B**) Female.

Figure 1 shows the cumulative CVD-specific mortality rates for different BMI categories in men and women with hypertension. In each BMI category, CVD-specific mortality increased with a decrease in BMI in both men and women (log-rank test, *P* < 0.001).

**Figure 2:**
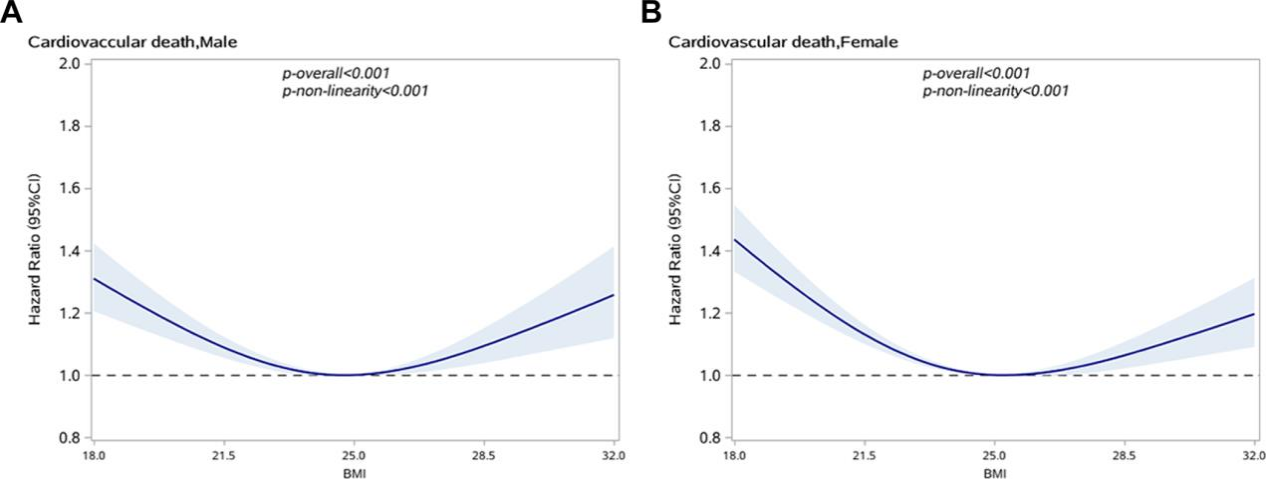
**Association between BMI and CVD specific mortality in people with hypertension by sex.** (**A**) Male; (**B**) Female.

A U-shaped relationship between BMI and CVD-specific mortality in hypertensive subjects was observed, as shown in Figure 2. Taking a BMI of 25 kg/m^2^ as a reference, men with BMI≤22.46 or ≥29.34 kg/m^2^ and women with BMI≤22.03 or ≥29.59 kg/m^2^ have significantly increased risk of CVD-specific death. By using 25kg/m^2^ as the reference value, the risk of death from CVD in men near the first percentile of BMI (17.65 kg/m^2^) increased by 34% (HR: 1.34, 95% CI: 1.23–1.47) and the risk of men near the 80th percentile of BMI (26.47 kg/m^2^) increased by 4% (HR: 1.04, 95% CI, 1.01–1.06), as shown in Table 3. Similarly, the risk of death from CVD in women near the first percentile of BMI (17.58 kg/m^2^) is increased by 47% (HR: 1.47, 95% CI, 1.36–1.59), and the risk of women near the 80th percentile BMI (26.67 kg/m^2^) increased by 3% (HR: 1.03, 95% CI, 1.01–1.05).

**Table 3:** Hazard ratios and 95% confidence intervals for BMI in association with CVD-specific mortality in people with hypertension of different sex.

**BMI**	**Male**					**Female**				
	**Value**	**HR**	**95%CI**	**Patients at risk, n**	**Mortality/100,000 person-y**	**Value**	**HR**	**95%CI**	**Patients at risk, n**	**Mortality/100,000 person-y**
10th percentile	20.76	1.13	1.09-1.18	9 318	1 589	20.28	1.23	1.18-1.29	1.23	1.18-1.29
20th percentile	21.97	1.08	1.05-1.11	9 819	1 110	21.48	1.14	1.11-1.18	1.14	1.11-1.18
30th percentile	22.72	1.04	1.02-1.06	10 462	976	22.43	1.08	1.06-1.10	1.08	1.06-1.10
40th percentile	23.45	1.01	0.99-1.02	9 991	892	23.23	1.02	1.00-1.03	1.02	1.00-1.03
50th percentile	24.22	1.00	1.00-1.01	9 640	790					
Reference	25.00	1.00				25.00	1.00		1.00	
60th percentile	24.77	0.99	0.99-1.00	9 475	769	24.65	0.99	0.99-1.00	0.99	0.99-1.00
70th percentile	25.61	1.02	1.01-1.03	10 405	714	25.48	1.01	1.00-1.02	1.01	1.00-1.02
80th percentile	26.47	1.04	1.01-1.06	10 089	674	26.67	1.03	1.01-1.05	1.03	1.01-1.05
90th percentile	27.77	1.07	1.03-1.12	9 908	623	28.13	1.06	1.02-1.10	1.06	1.02-1.10

Abbreviations: CVD: cardiovascular disease; BMI: body mass index; CI: confidence interval; HR: hazard ratio.

*Adjusted for sex, age at registry, systolic blood pressure, diastolic blood pressure, concomitant diabetes, family history of diabetes, family history of hypertension, family history of stroke, family history of CVD, smoking, drinking and physical activity.

**Figure 3:**
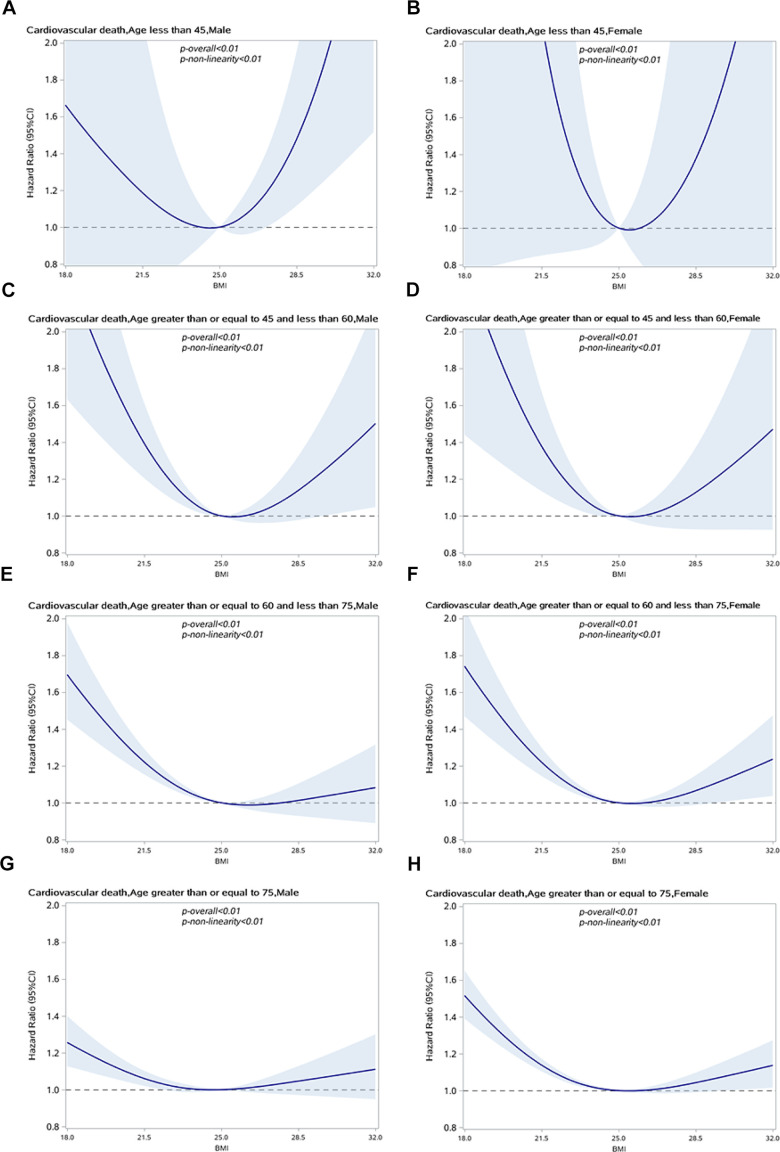
**Association between BMI and CVD specific mortality in people with hypertension by sex and age group.** (**A**) Male, age<45; (**B**) Female, age<45; (**C**) Male, 45<=age<60; (**D**) Female, 45<=age<60; (**E**) Male, 60<=age<75; (**F**) Female,60<=age<75; (**G**) Male, age>70; (**H**) Female, age>70.

We also performed a stratified analysis of age (<45, 45–59 years, 60–74 years, and ≥75 years) to identify the changes in the relationship between BMI and CVD-specific mortality in different age groups. The association between BMI and CVD-specific mortality was attenuated in men under 45 years and over 60 years and in women under 60 years, as shown in Figure 3. In older age groups, the risk of CVD-specific mortality for overweight and obese individuals was lower than in normal BMI individuals. The results remained unchanged in sensitivity analysis when excluding people who died within 2 years of enrollment, as shown in Supplementary Figure 1.

## DISCUSSION

We found a U-shaped relationship between BMI and the risk of CVD-specific mortality among patients with hypertension in this community-based retrospective cohort study in Shanghai.

An intervention study in a hypertensive population identified an increased risk of cardiovascular mortality in both thin and moderate to-severely obese individuals with hypertension and left ventricular hypertrophy in Europe [15]. Among patients with coronary heart disease, overweight and obese patients have lower all-cause mortality and CVD-specific mortality than normal weight patients [17]. Studies assessing the association between BMI and CVD-specific mortality in Chinese hypertensive patients have been limited, and the results have been inconsistent. A prospective cohort study conducted in Beijing involving 2,535 hypertensive patients aged 40–91 found a U-shaped relationship between BMI and CVD death [7]. However, the sample size of this study was insufficiently powered, and there may be unaccounted confounding factors such as physical activity and dietary factors that bias the results of the study. A study based on community hypertension patients in Xinzhuang, Minhang District found that BMI was not associated with CVD death among elderly hypertensive patients aged 60 years or older [18]. The sample comprised 10,957 individuals and 242 cardiovascular deaths were documented. BMI was used as a categorical variable in the Cox regression to determine the risk of CVD death, and the HR was calculated for each category. However, in order to observe the nonlinear relationship between BMI and CVD-specific mortality, BMI was not considered to be a continuous variable.

Since CVD is a chronic condition, observed deaths from CVD require longer follow-up time. Compared with other studies of hypertensive patients, ours had a larger sample size, a longer follow-up period, and a wider range of age. We used BMI as a continuous variable, and incorporated restricted cubic splines to the model to explore the nonlinear relationship between BMI and CVD death. Moreover, the possible confounding factors included in our analysis were more comprehensive. Previous studies have suggested that age should be used as an effect modifier in the relationship between BMI and all-cause mortality [19, 20]. Our study showed that the association between BMI and CVD-specific mortality is different in hypertensive patients of different age groups, thus confirming the modifying effect of age. In older people, overweight and obesity are potential factors that reduce the risk of CVD death. Similar findings were found in patients with heart disease, which is consistent with the obesity paradox [21, 22].

Several biological mechanisms may underlie the observed associations. The evidence for the association between obesity and high cardiovascular risk is compelling [23, 24], largely because in most cases, obesity is accompanied by metabolic syndrome [25]. Obesity increases the workload of the heart, thereby leading to the development of left ventricular hypertrophy, which is associated with poor prognosis [26, 27]. Moreover, there is increasing evidence that severe obesity is associated with the activation of inflammatory mechanisms [28, 29] that increase vascular thromboxane receptor gene expression, as well as the level of fibrinogen. This in turn may accelerate the progression of cardiovascular disease. The circulating markers activated by inflammation are mainly manifested in the fat distribution in the center of the body, and the damage of central obesity to the target organs is greater than the damage associated with peripheral body fat distribution [30, 31]. Low body weight can be harmful to the human body in many aspects. Studies have shown that low body weight may increase the risk of cardiovascular events [8]. In the older population, low body weight is often accompanied by poor nutrition, which further leads to reduced physiological function and survival [32]. At the same time, cardiac cachexia and physical weakness are also related to low body weight in the older population [33]. Previous studies have demonstrated that the increased metabolic reserve in overweight patients have a protective effect [22, 34], and that overweight patients are more motivated in self-health management and medication [35]. These physiological reactions, biochemical reactions and behavioral changes can be regarded as an explanation for the obesity paradox.

Several strengths of our study warrant mention. Our retrospective cohort study design, sample size (n = 212,394), and death events (n = 14,029) were sufficient to obtain reliable study results. Cox proportional hazard models with restricted cubic splines (RCS) were used to test potential non-linear relationships with high statistical reliability. Stratified analysis allows the assessment of possible sex differences in order to rule out the confounding effects of age associated with BMI and mortality. There are also a few limitations to our investigation. First, the BMI of hypertensive patients was calculated by self-reported weight and height, collected at different times and locations, without uniform measurement. These assessments may be subject to recall bias. Second, when assessing the relationship between BMI and mortality, only baseline BMI was included. However, with the prolongation of the disease course or disease management, the BMI of hypertensive patients may change over time. Third, we did not collect information on waist circumference and body fat percentage, so the impact of central obesity and abdominal obesity on CVD-specific mortality could not be assessed. Fourth, the number of patients with BMI <18.5 kg/m^2^ or ≥ 30kg/m^2^ was relatively small in this study population, especially after stratifying by age, which further reduced the statistical power of the results.

Hypertensive patients are a large population with a high risk of CVD death. Regular follow-up management of hypertensive patients by community doctors is a basic public health service in China. Our results clarify the association between BMI and the risk of CVD-specific mortality, and provide an appropriate BMI range within which CVD-specific mortality is reduced in hypertensive patients of both sexes. It is helpful for community doctors to carry out targeted weight management of hypertension patients and make patients aware of the early warning signs of cardiovascular disease.

In conclusion, this study demonstrates a U-shaped relationship between BMI and CVD risk of death in male and female patients with hypertension. Our findings may provide scientific guidance for weight management in patients with hypertension.

## MATERIALS AND METHODS

### Study population

**Figure 4:**
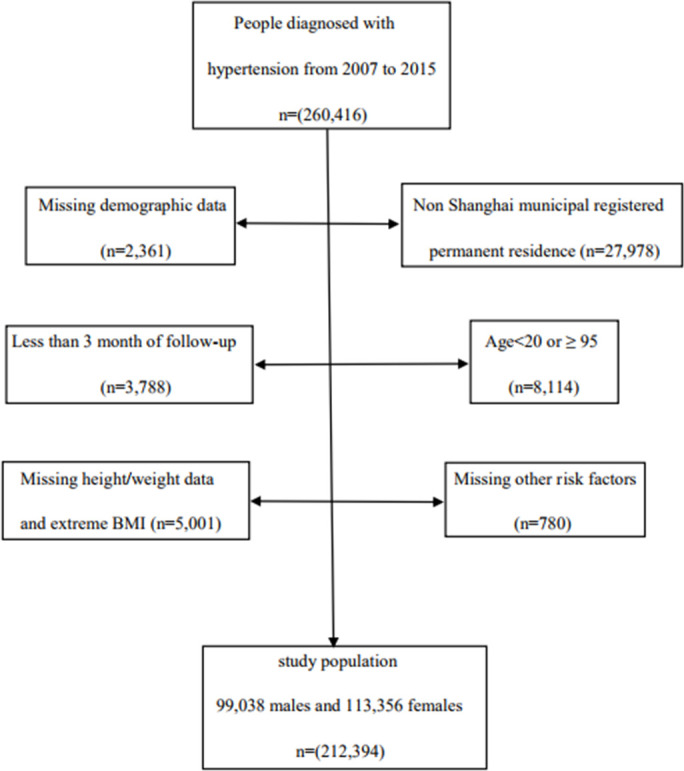
**Flow chart of study population.**

We conducted a population-based retrospective cohort study, which was based on the Minhang hypertension standardization management system. Since 2007, Minhang District, with 1 million permanent residents, has made standardized management of hypertensive patients a basic community health care service. During the period from 2007 to 2015, a total of 260,416 adults with hypertension were enrolled for standardized management and examined by a general practitioner in 13 community health service centers. All participants were followed up with respect to the three following factors: cardiovascular death, emigration, or December 31, 2018, whichever occurred first. Our study included 212,394 subjects aged 20–85 years, including 99,038 men and 113,356 women, after excluding patients with duplicate or abnormal ID numbers, abnormal names, missing or abnormal BMI, lack of information regarding factors related to risk, cases who were not permanent residents, and with less than 3 months of follow-up (Figure 4).

### Data sources

The diagnosis of hypertension is based on the 1999 WHO/ISH (World Health Organization/International Hypertension Federation) guidelines for the prevention and treatment of hypertension, wherein a blood pressure above 140/90 mmHg is defined as hypertension. After the participant sat quietly for five minutes, mercury sphygmomanometer was used to measure three blood pressure readings on different days according to a standard protocol. When these participants were registered in the Community Health Service Center, baseline information on demographic factors, daily exercise, smoking, drinking, family history of major chronic diseases, concomitant diabetes, self-reported standing height and body weight, and drugs used for hypertension treatment were collected and recorded in the electronic health record database in Minhang district of Shanghai, China [18, 36]. At the beginning of the follow-up period, information on the diagnosis of hypertension, weight, height, and other risk factors were verified for all participants by general practitioners. The staff of the Minhang District Center for Disease Control and Prevention randomly selected 10% of newly registered participants and verified the recorded information, including height and weight, via a telephonic interview.

### Body mass index

BMI was calculated as self-reported body weight in kilograms divided by squared self-reported standing height in meters. In this study, we used the BMI classification set by the WHO: underweight (< 18.5 kg/m^2^), normal weight (18.5–24.9 kg/m^2^), overweight (25.0–29.9 kg/m^2^), and obesity (> 30.0 kg/m^2^).

### Outcome

The occurrence of CVD-specific death was the end point of the study. The unique personal identification number assigned to all residents allowed accurate linkage to the Shanghai Vital Statistics. The outcome of interest, CVD-specific mortality, was identified using International Classification of Diseases (ICD-10) codes for the primary cause of death and were classified into disease groups of CVDs (ICD-10 codes I00-I99).

### Covariates

Potential confounders, including sex (male/female), age at registry (continuous), systolic blood pressure (continuous), diastolic blood pressure (continuous), concomitant diabetes (yes/no), family history of diabetes (yes/no), family history of hypertension (yes/no), family history of stroke (yes/no), family history of CVD (yes/no), smoking (everyday, sometimes, quit, never), drinking (Everyday, always, sometimes, never), and physical activity (everyday, > once per week, sometimes, never) were considered covariates for this study.

### Statistical analysis

Pearson’s chi-square test and Kruskal-Wallis test were used to analyze the characteristics of participants in different groups of BMI. We used the Kaplan-Meier method to compute cumulative incidence in different BMI groups of different sexes. Cox regression models were used to estimate HRs and assessed the association between different BMI groups and cardiovascular death among hypertensive subjects. The adjustment variables included were sex, age at registry enrollment, systolic blood pressure, diastolic blood pressure, concomitant diabetes, family history of stroke, family history of hypertension, family history of cardiovascular disease, family history of diabetes, smoking, drinking, and physical activity. We first evaluated the PH hypothesis of each variable in the Cox proportional hazards model, and used it as a three-section-restricted RCS by fitting the interaction between each variable and time [37]. Variables tested against the PH hypothesis included physical activity, diabetes comorbidity, and familial history of hypertension. After adjusting for variables that did not meet the PH hypothesis, according to the BMI category, HRs and 95% CIs of CVD-specific mortality were calculated. In addition, we used BMI as a continuous variable to estimate the potential curvilinear correlation between BMI and cardiovascular mortality by using RCS with decile intervals as fixed knots. In this process, two statistical tests were performed. A regression coefficient applied to the null hypothesis, that is, the linear and nonlinear terms of the factor are equal to zero – in other words, no correlation. Another is used for the regression coefficient of the nonlinear term, where *P* <0.05 represents the nonlinear association. Taking a BMI of 25.0 kg/m^2^ as a reference, the Cox regression model with RCS function was used to estimate the multivariate adjusted HRs and 95% CIs, and the associations were visually illustrated. We then conducted a stratified analysis based on sex and age. After excluding patients who died within 2 years of enrollment, sensitivity analysis was performed to reduce the influence of reverse causality.

All analyses were conducted using SAS (version 9.4; SAS Institute, Cary, NC, USA). All statistical tests were two-tailed, and *P* value of 0.05 was considered statistically significant.

### Ethics approval and consent to participate

The study was approved by the Institutional Review Board of center for disease control and prevention in Minhang District, Shanghai (NO: EC-P-2019-009). Informed consent from participants involved in the study was waived due to the fact that anonymized data compiled from electronic medical records was applied in the study.

### Data availability

The datasets generated and analyzed in the study are not publicly available but are available from the corresponding authors on reasonable request.

## Supplementary Material

Supplementary Figure 1
