# Supplementary material for: Association between body mass index and risk of cardiovascular disease-specific mortality among adults with hypertension in Shanghai, China
Source: Aging (Albany NY). 2021 Feb 17;13(5):6866–77. doi: 10.18632/aging.202543 (PMC7993713; doi:10.18632/aging.202543)
Supplement: Supplementary Figure 1 [file aging-13-202543-s001.pdf]

SUPPLEMENTARY FIGURE

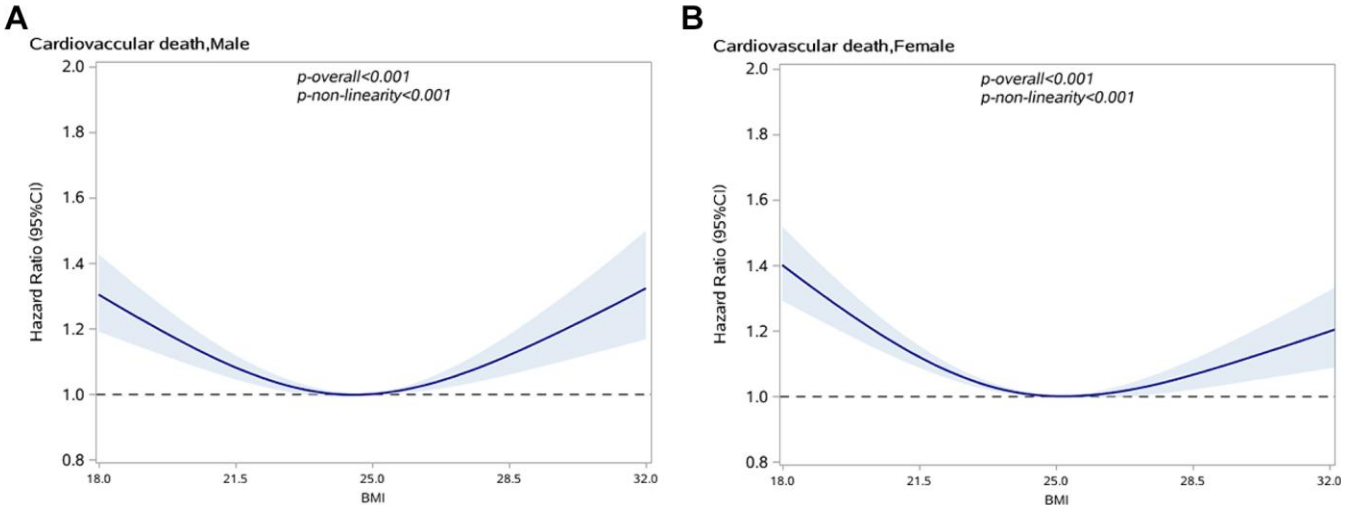

Supplementary Figure 1. Association between BMI and the risk of CVD specific mortality in people with hypertension by sex, allowing for non-linear effects. (**A**) Male; (**B**) Female.
